# Immunomodulation of the Vaginal Ecosystem by *Ligilactobacillus salivarius* CECT 30632 Improves Pregnancy Rates among Women with Infertility of Unknown Origin or Habitual Abortions

**DOI:** 10.3390/nu15020362

**Published:** 2023-01-11

**Authors:** Leónides Fernández, Irma Castro, Rebeca Arroyo, Claudio Alba, David Beltrán, Juan M. Rodríguez

**Affiliations:** 1 Department of Galenic Pharmacy and Food Technology, Complutense University of Madrid, 28040 Madrid, Spain; 2 Department of Nutrition and Food Science, Complutense University of Madrid, 28040 Madrid, Spain; 3 Centro de Diagnóstico Médico, Ayuntamiento de Madrid, 28006 Madrid, Spain

**Keywords:** *Ligilactobacillus salivarius*, habitual abortion, infertility, probiotics, TGF-β, VEGF, safety

## Abstract

In this study, the probiotic potential of *Ligilactobacillus salivarius* CECT 30632 was assessed, including properties specifically related with gynecological targets. This strain displayed co-aggregative and antimicrobial activity against a wide spectrum of vaginal pathogens while being respectful with the growth of vaginal lactobacilli. The strain produced a high concentration of lactic acid and displayed α-amylase activity when assayed in vitro. It showed a noticeable survival rate after exposition to conditions similar to those present in the human digestive tract and was adhesive to both vaginal and intestinal cells. Subsequently, their capacity to increase pregnancy rates among women with habitual abortion or infertility of unknown origin was studied. Administration of *L. salivarius* CECT 30632 (~9 log_10_ CFU) daily for a maximum of six months to these women was safe and led to a successful pregnancy rate of 67.5% (80% and 55% for women with repetitive abortion and infertile women, respectively). Significant differences in Nugent score, vaginal pH, and vaginal concentrations of lactobacilli, TGF-β, and VEFG were observed when the samples collected before the intervention were compared with those collected after the treatment among those women who got pregnant. Therefore, this strain can modulate the vaginal ecosystem and lead to better fertility outcomes.

## 1. Introduction

Dominance of the genus *Lactobacillus* species and, particularly, of those belonging to the species *L. crispatus*, *L. jensenii*, *L. iners,* and *L. gasseri*, which may account for even higher than 90% of the vaginal microbiota, is a characteristic feature of the vaginal microbiota of fertile women under physiological conditions [1,2]. Several factors may alter its composition [3] and any deviation from the paradigm of a healthy vaginal microbiome (*Lactobacillus* dominance, low diversity) is frequently linked to negative gynecological and obstetric outcomes, including infertility [4,5,6]. Similarly, bacteriome studies focused on endometrial samples have shown that a low percentage of *Lactobacillus* sequences is a common signature among infertile women while the contrary is associated with a higher implantation success [7,8]. As a consequence, there is a growing awareness of the importance of the female genital tract microbiota for reproductive success [9,10].

Probiotics have been postulated as a method to improve the outcomes achieved by assisted-reproduction technologies (ARTs) [11]. In fact, empiric use of commercial probiotic products is increasingly prescribed as an adjuvant treatment for women with infertility of unknown cause [10], despite that there is no scientific or clinical evidence to support its usefulness for this target. In a previous work, our group found that oral administration of *Ligilactobacillus salivarius* CECT 5713 was able to modify immunological and microbiological parameters in the vaginal ecosystem of women with a previous history of reproductive problems, leading to a rate of term pregnancies of 56% among the participants [12]. In this study, another *L. salivarius* strain previously isolated from a woman with a long record of genitourinary tract health and a successful reproductive history was characterized for a wide variety of general and vaginal-related probiotic properties; subsequently, it was orally administered to women with habitual abortion (implantation failure) or infertility in order to elucidate its safety and its efficacy in improving fertility outcomes. Before the initiation of the trial, a variety of vaginal parameters (Nugent score, pH, microbiological and immunological profiles) were studied, and the data compared with those obtained from healthy fertile women. At the end of the trial, the same parameters were assessed among the treated women.

## 2. Materials and Methods

### 2.1. Ligilactobacillus Salivarius CECT 30632

The strain CECT 30632 was isolated in the frame of a previous study [13] from the vaginal exudate of a normoweight woman who met the following criteria: (a) no history of genitourinary tract infections and a low record of antibiotic use; (b) two previous term pregnancies without any complication; (c) abundant presence of lactobacilli in the vaginal sample (>6 log_10_ colony-forming units (cfu)/swab) after culturing on MRS plates; (d) absence of detection of chlamydias, trichomonas, *Gardnella vaginalis, Ureaplasma* spp. *S. agalactiae*, *Mycoplasma* spp., and *Candida* spp. (or any other yeast) in the vaginal samples; and (e) negative (blood screening) to human immunodeficiency viruses (HIV), cytomegalovirus, human papillomavirus (HPV), gonorrhea, and syphilis. In addition, strain-related criteria used to select strain CECT 30632 were its capacity to grow (≥1 × 10^8^ cfu/mL) in routine broth medium (MRS broth) after incubation (16 h at 37 °C).

The strain was identified as *Ligilactobacillus salivarius* using Matrix Assisted Laser Desorption Ionization-Time of Flight (MALDI-TOF) mass spectrometry (Bruker, Germany). The European Food Safety Authority (EFSA) includes this species among those with the QPS (qualified presumption of safety).

### 2.2. Assessment of Probiotic Properties of L. salivarius CECT 30632

The general scheme for the assessment of the potential probiotic features of *L. salivarius* CECT 30632 is essentially the same that has already been published for *L. salivarius* CECT9145 [13]. The tests were performed in triplicate. The antimicrobial activity of the strain against vaginal pathogens was performed using an overlay method [14,15]. The indicator strains included two strains of *Candida parapsilosis,* two of *Candida glabrata*, three strains of *Candida albicans*, three of *Streptococcus agalactiae*, five of *G. vaginalis*, and two of *Ureaplasma urealyticum.* These strains were isolated from women with cervico-vaginal infections (our own collection). The bacteriocinogenic ability of the strain was evaluated using an agar diffusion assay as described [15], using the same indicator microorganisms. Parallelly, potential co-aggregation between *L. salivarius* CECT 30632 and such vaginal pathogens was assessed as described previously [16].

The ability of *L. salivarius* CECT 30632 to adhere to vaginal and intestinal (Caco-2 and HT-29) epithelial cells was evaluated following methods reported previously [15,16], using a highly adhesive strain (*L. salivarius* CECT9145) as a positive control [13]. Adhesion of the probiotic strain to porcine mucin was assayed as described [17].

L- and D-lactic acid concentrations in the MRS supernatants of *L. salivarius* CECT 30632 (obtained after an incubation at 37 °C for 16 h) were assayed enzymatically using the Roche Diagnostics (Mannheim, Germany) kit. Their pH values were also determined. Hydrogen peroxide production by the strain was measured as described [15]. The α-amylase activity of the probiotic strain was studied because of the beneficial implications of this enzyme for strains aimed to vaginal applications [18]. Initially, this activity was evaluated qualitatively using the procedure of Padmavathi et al. [19]. Later, the α-amylase enzymatic activity associated with *L. salivarius* CECT 30632 cells was quantified using a specific kit (Kikkoman Co., Tokyo, Japan) as described previously [20].

Since the administration route chosen for the fertility trial was oral delivery, the capability of *L. salivarius* CECT 30632 to survive after exposition to oral and gastrointestinal-like conditions was evaluated. For this purpose, the methodology proposed by Marteau et al. [21], and modified by Martín et al. [22], was selected.

Safety assessment of probiotic strains must include their susceptibility to antibiotics. In this study, susceptibility of *L. salivarius* CECT 30632 to these antimicrobials was evaluated using the E-test system (Biomerieux) and the cut-off values proposed for this species by EFSA [23]. In addition, the strain was searched for haemolysis when growing on horse blood agar medium [13], for biogenic amines biosynthesis (putrescine, cadaverine, tyramine and histamine) [24], and for its potential for gastric mucine (HGM; Sigma) degradation [25]. EFSA recommends oral toxicity testing of foods, including food supplements consisting of or isolated from microorganisms, using outbred Wistar or Sprague-Dawley rats or CD-1 mice. In this study, assessment of acute and repeated dose (4-weeks) oral toxicity of *L. salivarius* CECT 30632 was evaluated in Wistar rats as previously described [13]. These in vivo assays were performed following the European Union guidelines (EC Council Regulation No. 440), and after obtaining the authorization of the UCM’s Ethical Committee on Animal Research (protocol 240111). Clinical and behavior observations, histopathological analysis, hematology analysis, and blood biochemistry were performed as reported previously [26]. The method described by Lara-Villoslada et al. [27] was employed to assess total liver glutathione (GSH) and potential systemic translocation (spleen, liver, or blood) of the probiotic strain.

### 2.3. L. Salivarius CECT 30632 to Increase Fertility-Related Outcomes: A Pilot Clinical Trial

In this trial, 74 women were recruited and classified in 4 different groups. RA (repetitive abortion) women (*n* = 20) had experienced ≥ 3 miscarriages within the first 12 weeks of pregnancy. INF (infertile) women (*n* = 20) were characterized by their inability to conceive after, at least, three ART attempts, including in vitro fertilization (IVF). The non-pregnant control group (NPC; *n* = 14) was integrated by non-pregnant women who were the mothers of ≥2 healthy children delivered at term after normal pregnancies. This group was used to compare their demographic, anthropometric, and medical data, and their microbiological and immunological vaginal profiles with those of the women belonging to the previous two groups. Finally, the pregnant control group (PC; *n* = 20) included healthy pregnant women with natural pregnancies (without the use of assisted reproduction techniques or treatments), who were randomly recruited among women recently diagnosed as pregnant by the same gynecologist. They were employed to compare some health and safety-related parameters, including systolic and diastolic blood pressure in each pregnancy trimester, gestational age at birth or total weight gain during pregnancy, with those of the RA and INF women who became pregnant in the assay.

Exclusion criteria for the RA and INF groups included intention to be the recipients of ART therapies during the assay, suffering from antiphospholipidic syndrome (to avoid the use of salicylic acid and/or heparin during the trial), and the use of antibiotics, probiotics, and/or hormonal therapy during the 4 weeks before recruitment and sampling.

At day 0 (any of the first three days after ovulation), a wide variety of anthropometric, demographic, and health-related data were recorded from RA, INF, and NPC women. Parallelly, the vaginal pH was measured, and the following samples were collected: (a) a vaginal swab for Nugent score assessment, and (b) a cervicovaginal lavage (CVL) for the microbiological (culture-based and PCR-based analyses) and immunological assays. CVL samples were obtained, processed, and stored as reported previously [28]. From day 0, RA and INF women ingested a sachet containing the freeze-dried test strain (*L. salivarius* CECT 30632) at a concentration of approximately 9 log_10_ CFU, daily for 6 months or until a diagnosis of pregnancy. Then, vaginal pH was recorded and a vaginal swab and a CVL sample were obtained again. Ingestion of the probiotic strain was continued for the first 15 weeks of pregnancy if an RA or INF woman became pregnant. Compliance with intake of the test product was recorded through daily diaries.

The culture-based microbiological analysis of the CVL samples was carried out using the media and incubation conditions described previously [12]. The media included MRS agar (Oxoid, Basingstoke, UK) supplemented with horse blood (5%) or L-cysteine (2.5 g/L) and CHROMagar StrepB, Mac Conkey, Columbia Nalidixic Acid (CNA), Sabouraud Dextrose Chloramphenicol, Mycoplasma, and Gardnerella agar plates (BioMerieux, Marcy l’Etoile, France). The isolates were identified by either 16S ribosomal RNA (rRNA) gene sequencing [29] or MALDI-TOF mass spectrometry (Bruker, Germany).

DNA was extracted from aliquots (1 mL) of the CVL samples [30] and employed for detection and quantification of *L. salivarius* DNA by quantitative PCR (qPCR) using the procedure described by Harrow et al. [31] and modified by Fernández et al. [12]. Using such a technique, threshold cycle (Ct) values for *L. salivarius* DNA oscillate between 15.29 and 20.07 for a DNA concentration ranging from 2.0 ng to 0.2 pg (R^2^ > 0.99). DNA from *Lactiplantibacillus plantarum* MP02 and *Limosilactobacillus reuteri* MP07 was used as negative controls (Ct ≥ 39).

The immunological analysis of the CVL samples included the measurement of the concentrations of a wide variety of cytokines, chemokines, and growth factors (IL2, IL4, IL5, IL6, IL7, IL8, IL9, IL10, IL12, IL13, IL15, IL17, IL6, VEGF, TNFα, RANTES, PDGF-BB, MIP1β, MIP1α, MCP1, IFNγ, GCSF, GMCSF, basic FGF, eotaxin, IL17, IL16, IL15, IL13, IL12, IL10, IL9, IL8, IL7, IL6, IL5, IL4, IL2, IL1β, and IL1ra,) using the Bio-Plex Pro™ Human Cytokine 27-plex Assay (Bio-Rad, Hercules, CA, USA) and the Bioplex 200 platform (Bio-Rad). In addition, the concentrations of TGF-β 1 and TGF-β 2 were determined using the kits RayBio^®^ Human TGF-β 1 and Human TGF-β 2 ELISA (RayBiotech, Norcross, GA, USA).

Health and safety parameters were recorded from women of the NPC group and, also from those of the RA and INF groups that became pregnant during this assay. Checks during pregnancy included assessment of systolic and diastolic blood pressure during each trimester measured by a nurse using a standard procedure. Total gestational weight gain was defined as the difference between the weight measured immediately before delivery and the pre-pregnancy weight.

Adverse events (AEs) and severe adverse events (SAEs) were also recorded (MedDRA version 17.1). Potential development of gestational diabetes and preeclampsia was carefully assessed.

The protocol was approved by the Ethical Committee of Clinical Research of Hospital Clínico San Carlos (Madrid, Spain; protocol 10/017-E). Written informed consent was obtained by all the recruited women. The register of the trial is available (ref. number NCT04446572) in the ClinicalTrials.gov database.

### 2.4. Statistical Analysis

Microbial counts data were recorded as log_10_ CFU/mL. The Shapiro–Wilks test was used to analyze the normality in the distribution of the data. Those quantitative variables that followed a normal distribution were expressed as means and 95% confidence intervals (CI) or standard deviations (SD) while those that were not normally distributed were expressed as medians and interquartile ranges (IQR). Qualitative values were recorded as total number of events and/or their percentages. The means of the experimental groups were compared using one-way ANOVA tests while Scheffé *post hoc* tests served for identifying those pairs of means that were different from a statistical point of view. One-way ANOVA repeated measures tests were employed to analyze the effect of the intervention on the vaginal-related parameters in the INF and RA groups. The Fisher’s exact probability test, or the Freeman–Halton extension of the Fisher exact probability test for a 2 × 3 contingency table, was employed in order to compare proportions and frequencies. In the case of non-parametric analyses, Kruskal–Wallis and Wilcoxon–Mann–Whitney tests were used to assess differences between groups and, when required, the Bonferroni’s correction for multiple comparisons was performed. Statgraphics Centurion XVIII version 18.1.06 (Statgraphics Technologies, Inc., The Plains, VA, USA) or the R environment (version 3.5.1; R-project, http://www.r-project.org; accessed on 3 September 2022) and *ggplot2* were employed for the statistical analysis of the data included in this work. The level of significance was set at *p* < 0.05.

## 3. Results

### 3.1. Assessment of the Probiotic Potential of L. salivarius CECT 30632

*L. salivarius* CECT 30632 displayed inhibitory activity and co-aggregation ability against all the vaginal pathogens used as indicator microorganisms in this work (Table 1). Co-aggregation between the probiotic strain and the *Candida* and *G. vaginalis* strains was particularly intense (Table 1). Bacteriocin activity was not detected against the indicator organisms used in this study.

The adherence of *L. salivarius* CECT 30632 to HT-29, Caco-2 and vaginal cells was high since it showed means (±SD) of 352.7 (±64.5), 343 (±51), and 897.2 (±190.1) adhered lactobacilli cells in 20 random microscopic fields, respectively. These values were similar to those achieved by *L. salivarius* CECT9145, the highly adhesive strain used as a control: 342.9 (±69.4), 904.4 (±229.7), and 333 (±56), respectively. Adhesion of *L. salivarius* CECT 30632 to porcine mucin (11.3% [±1.6] of retained fluorescence) was also very similar to that showed by *L. salivarius* CECT 9145 (10.7% (±1.8)).

L-lactic acid production by *L. salivarius* CECT 30632 when growing in MRS broth for 16 h at 37 °C was 10.29 mg/mL (±0.53), which corresponded with a mean pH value of 3.96. The production of this lactic acid isomer by a high acidifying strain (*L. salivarius* CECT9145) was 10.12 (±0.47) (pH: 4.00). No detectable amounts of D-lactic acid were present in the culture supernatants of *L. salivarius* CECT 30632. The amount of hydrogen peroxide produced by *L. salivarius* CECT 30632 was approximately 0.7 μg/mL.

The colonies of *L. salivarius* CECT 30632 produced a clearance halo (1.9–2.1 mm) when the growth medium was flooded with iodine solution, indicating amylase production. Subsequently, a noticeable level of α-amylase activity (0.80–0.84 U/mL) was detected after 16 h of incubation, when the concentration of the strain was 8.5–8.9 log_10_ CFU/mL. Such activity remained in the culture supernatants for, at least, 48 h.

The viability of *L. salivarius* CECT 30632 after exposition to oral and gastrointestinal-like conditions was ~62.5%, a value comparable to that achieved by *L. salivarius* CECT9145 (64.1%). The later strain was selected as a control because of its high viability after being exposed to the same conditions.

The MIC values of *L. salivarius* CECT 30632 for the 16 antibiotics tested in this study using the E-test procedure revealed that the strain was sensitive to most of them, with MIC values that are acceptable according to the breakpoints proposed by EFSA [23] (Table 2). The strain showed resistance to kanamycin and vancomycin, but this is an intrinsic feature of the *L. salivarius* species (Table 2).

Concerning other safety-related properties, *L. salivarius* CECT 30632 lacked haemolytic activity, and neither degraded gastric mucin nor produced biogenic amines. In the rat-model, all animals survived to the toxicity assays. All of them showed a development (size, weight) that was within the normal parameters for this species and age. No differences were detected between animals of the treated and control groups (including satellite groups) in any parameter, including, body weight gain, organs’ weight, haematological and clinical chemistry, or behaviour, at the end of the experimental period. The concentrations of liver GSH were similar in the control and treated groups (9.42 ± 1.32 versus 9.34 ± 1.41 mmol/g, *p* > 0.4). The strain was isolated from the feces (4.84 and 7.94 log_10_ cfu/g) and vaginal samples (3.53 and 6.29 log_10_ cfu/swab) collected from the rats of the probiotic groups at the end of the treatment. In contrast, it was not possible to detect any colony belonging to the species *L. salivarius* in any sample from the control group.

### 3.2. Pilot Clinical Trial: General Description of the Recruited Women

Table 3 shows the characteristics of the 54 women of the RA, INF, and NPC groups. There was no difference between the three groups regarding the mean values of body weight and height. In relation to the age, the means (95% CI) were 34.6 years (33.5–35.8), 39.5 (38.5–40.9), and 37.95 (36.92–38.98) in the NPC, RA, and INF groups, respectively (Table 3). The statistical analysis revealed that women in the INF and RA groups were significantly older than NPC participants (*p* = 0.001; one-way ANOVA). Differences between the NPC women, on one side, and RA and INF women, on the other side, were also detected regarding the number of previous episodes of urinary tract and vaginal infections, which was higher in the last two groups than in the NPC group (*p* = 0.013 and *p* = 0.024, respectively; Fisher exact probability tests) (Table 3; Supplementary Figure S1). The use of antibiotics during infancy and adulthood, defined as receiving at least four annual treatments due to recurrent infections, was also higher among RA and INF women than among NPC women (*p* < 0.001 and *p* = 0.016) (Table 3; Supplementary Figure S1). However, the rates of gastrointestinal, respiratory, and skin infections did not differ among these three study groups (Table 3).

### 3.3. Pilot Clinical Trial: Vaginal-Related Parameters at Baseline

The values of vaginal pH in the NPC group (4.53; range 4.38–4.68) statistically differed from those obtained from RA (5.74 [5.53–5.94] and INF 6.03 [5.88–6.18] women (*p* = 0.000; one-way ANOVA). Nugent scores within the RA (6.55 [5.99–7.11]) and INF (6.40 [5.90–6.90]) groups were higher than that obtained from the NPC group (1.79 [1.27–2.30]; *p* < 0.001; one-way ANOVA) (Table 4). The concentrations of TGF-β1 (4.83 [4.65–5.01] pg/mL), TFG-β2 (3.22 [3.10–3.34] pg/mL), and VEFG (406.0 [322.0–490.0] pg/mL) in the CVL samples of the NPC group were statistically higher than those present in the CVL samples of RA and INF women (Table 4). Regarding the rest of immune factors analyzed in this trial, there was a high degree of inter-individual variability, and no differences could be detected when samples from the three groups were compared (data not shown).

**Figure 1:**
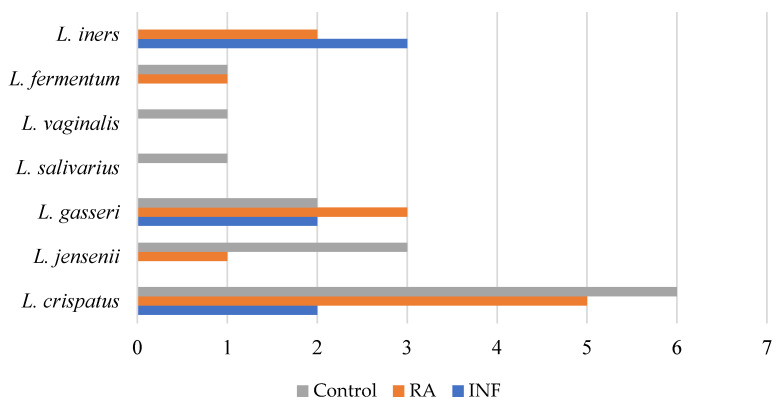
Lactobacilli species isolated from the vaginal samples of the women belonging to either the NPC group, the RA group, or the INF group.

Lactobacilli were isolated in the vaginal samples of all the women (100%; *n* = 14) of the NPC group. The mean (95% CI) of the vaginal lactobacilli count in this group was 7.33 (7.15–7.46) log_10_ CFU/mL. In contrast, lactobacilli were detected only in 60% and 35% of the samples collected from the RA and INF groups, respectively (*p* < 0.001; Fisher exact probability tests). Additionally, the mean vaginal lactobacilli counts in the lactobacilli-positive women of the RA and INF groups were 2.62 and 2.75 log_10_ units lower, respectively, than those observed in the NPC group. The profile of *Lactobacillus* species in the NPC group differed from that observed in the RA and INF groups (Figure 1). Up to six different species could be isolated from the samples of the NPC group, including *L. vaginalis*, *L. salivarius*, *L. fermentum*, *L. gasseri*, *L. jensenii,* and *L. crispatus.* In contrast, the RA and INF groups displayed narrower profiles and two of the species cited above *(L. vaginalis* and *L. salivarius*) could not be isolated. *L. crispatus* was the species that reached the highest concentrations in six (43%) of the NPC group samples, in five (25%) samples from the RA group, and in only two (10%) samples from the INF group. *L. iners* could not be detected from any of the NPC samples but it was isolated from five of the 19 lactobacilli-positive samples of the RA and INF groups. The species *L. salivarius* was isolated (7.3 log_10_ CFU/mL) and PCR detected (7.29 log_10_ copies/mL) only from one sample belonging to a woman of the NPC group. RAPD genotyping revealed that the strain isolated from that woman was different from *L. salivarius* CECT 30632 (results not shown).

### 3.4. Pilot Clinical Trial: Pregnancy Effectiveness and Successful Pregnancy Effectiveness

Daily intake of *L. salivarius* CECT 30632 for up to six months to the 40 women of the RA and INF groups led to 27 pregnancies (67.5% of pregnancy effectiveness; 95% CI: 53–82% (Table 5), including 25 term pregnancies (gestational age ≥ 38 weeks) and two abortions (which happened in the first 12 weeks of pregnancy). This represents a successful pregnancy effectiveness of 62.5% (95% CI: 48–78% (Table 5)).

The highest rate of success (one abortion and 15 full term pregnancies) was observed among women of the RA group (Table 5) although the rate among women of the INF group was also relevant (one abortion and 10 full term pregnancies).

### 3.5. Pilot Clinical Trial: Secondary Outcomes in the RA and INF Groups

There were statistically significant differences related to some vaginal parameters between those RA women that had a successful pregnancy after the trial (*n* = 15) and those who did not (*n* = 5). The vaginal pH of RA women who got a term pregnancy was 1.18 units lower than that measured in those who did not (*p* < 0.01; one-way ANOVA). Similarly, there was a mean (95% CI) reduction of 4.2 (4.68–3.26) units in the Nugent score of the RA women who got pregnant; in contrast, the reduction was significantly lower (1.2 [−1.43–−0.75] units) among the remaining women of the same group (*p* < 0.01; one-way ANOVA) (Table 6; Supplementary Figure S1).

Both subgroups of RA women (successful pregnancy or not) also differed in the vaginal concentrations of some immune factors after the trial. While no modification was observed in their vaginal concentrations of VEGF, TGF-β1, and TGF-β2 in those without a pregnancy, there was a significant increase (*p* < 0.001; one-way repeated measures ANOVA) in the means (95% CI) of such growth factors in those who became pregnant. The increases were of 306.40 (233.76–449.93) pg/mL, 1.59 (1.31–2.15) pg/mL, and 1.36 (1.17–1.73) pg/mL, respectively (Table 6). It must be highlighted that even at baseline (day 0), there were differences in the concentration of these three growth factors between RA women that became pregnant and those that did not (Table 6).

**Figure 2:**
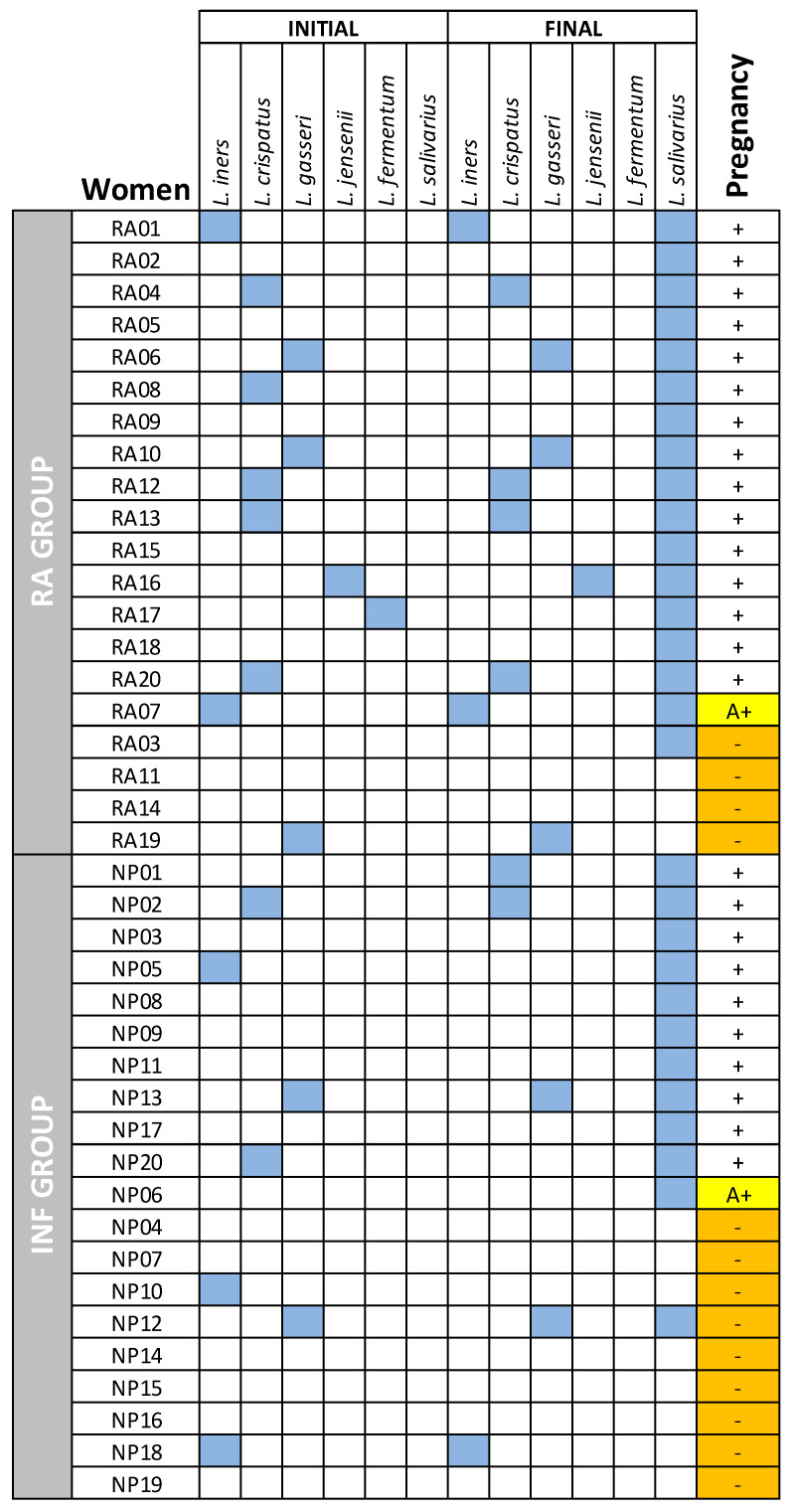
*Lactobacillus* species isolated from vaginal samples of women of the RA and INF groups before and after the trial. The pregnancy outcome of each woman is shown in the last column: -, no pregnancy; +, full-term pregnancy; A+, abortion. Isolation of a given species from a sample is indicated as a blue box.

The oral administration of the probiotic strain also resulted in a significant increase in the vaginal *Lactobacillus* concentration (mean [95% CI]: 3.12 [2.51–4.33] log_10_ CFU/mL) in RA women that got pregnant; in contrast, no significant changes were observed in the RA subgroup that did not become pregnant (Table 5). Figure 2 presents the different *Lactobacillus* species that were isolated from the vaginal samples obtained at baseline and after the trial from each woman. The biggest difference in the vaginal *Lactobacillus* patterns was the isolation of *L. salivarius* in most RA women (17/20) after the trial. Presence of *L. salivarius* specific DNA was detected by qPCR in the vaginal samples of all RA women that got a pregnancy. The mean (95% CI) in this subgroup was 7.81 (7.52–8.39) log_10_ copies/mL; however, *L. salivarius* DNA was only detected in half of the samples provided by RA women who did not get pregnant and, when *L. salivarius* DNA was detected, its concentration [mean (95% CI) = 2.25 (1.50–3.73) copies/mL] was statistically lower (Table 6).

After the trial, the values of vaginal pH and the Nugent scores, expressed as means (95% CI), decreased in all the women of the INF group (*p* < 0.05; one-way repeated measures ANOVA), but the change in both parameters was bigger in those that did get pregnant than in those that did not (Table 7; Supplementary Figure S1). The decrease in pH in INF women that became pregnant was −1.41 (−1.59–1.23) but only −0.28 (−0.43–0.13) in those who did not have a pregnancy. Similarly, the decrease in Nugent score in INF women that became pregnant was −4.2 (−4.90–3.50) but only −0.9 (−1.44–0.36) in those who did not have a pregnancy (Table 7; Supplementary Figure S1).

Both INF subgroups (successful pregnancy or not) also differed in the vaginal concentrations of some immune factors after the trial. While no modification was observed in their vaginal concentrations of VEGF, TGF-β1, and TGF-β2 in those without a pregnancy, there was a significant increase (*p* < 0.001; one-way repeated measures ANOVA) in the means (95% CI) of such growth factors in those who became pregnant. The increases were of 472.3 (379.31–565.29), 2.32 (2.16–2.48) pg/mL, and 1.35 (1.26–1.44) pg/mL, respectively (Table 7). Similarly to the RA subgroups, differences in the concentration of these growth factors between INF women that became pregnant and those that did not were already present at baseline (Table 7).

The administration of the probiotic strain also resulted in a significant increase in the vaginal *Lactobacillus* concentration (mean [95% CI]: 6.70 [6.19–7.15] log_10_ CFU/mL) in all the INF women that got pregnant; in contrast, such effect was observed only in 30% of the INF subgroup who failed to get pregnant and, in such cases, the *Lactobacillus* concentration was significantly lower [5.4 (3.73–6.88) log_10_ CFU/mL] (Table 7). Figure 2 presents the different *Lactobacillus* species that were isolated from the vaginal samples obtained at baseline and after the trial from each woman. The biggest difference in the vaginal *Lactobacillus* patterns was the isolation of *L. salivarius* from all the samples of the INF women that became pregnant but only from one of the INF women that did not have a pregnancy. Presence of *L. salivarius* specific DNA was detected by qPCR in the vaginal samples of all the INF women that got a pregnancy. The mean (95% CI) in this subgroup was 7.05 (6.39–7.63) log_10_ copies/mL; however, *L. salivarius* DNA was only detected in 10% of the samples provided by INF women who did not get a pregnancy (Table 7).

Overall, the analysis of some vaginal parameters revealed that the vaginal pH, the Nugent scores, the VEGF, TGF-β1, and TGF-β2 concentrations, and the lactobacilli counts in women RA and INF who became pregnant after the trial were similar or closer to those found in the vaginal samples obtained from the NPC group, composed by healthy fertile women (Table 4).

### 3.6. Comparison of Other Health and Safety Parameters between PC Women and Those Who Got Pregnant from the RA and INF Groups

Oral administration of *L. salivarius* CECT 30632 was well tolerated by all the recruited women belonging to either the RA or the INF group. In total, 12 (60%) of subjects in the PC group reported ≥ 1 AE during their pregnancies, as compared to eight (40%) and nine (45%) of the pregnant women belonging to the RA and INF study groups, respectively. None of the reported AEs in the RA and INF groups was related to the probiotic product. No differences were found regarding the presence of gastrointestinal diseases although, overall, the self-reported bowel habit of the RA and INF pregnant women was better than that of pregnant women of the PC group, which was associated to a higher rate of constipation.

In the INF group, the mean systolic blood pressure (SBP) values for the first, second, and third trimesters (95% CI) were 114.5 (110.97–118.03), 114.8 (111.12–118.48), and 115.1 (111.41–118.79) mm Hg, respectively. The diastolic blood pressure (DBP) values were 71.10 (67.62–74.56), 70.70 (67.39–74.01), and 71.00 (67.99–74.00) mm Hg, respectively. No statistical differences were found between the three different trimesters for both SBP and DBP. In the RA group, the mean SBP values for the first, second, and third trimesters (95% CI) were 114.25 (110.37–118.13), 113.8 (110.35–117.25), and 113.8 (109.68–117.92) mm Hg, respectively. The DBP values were 72.625 (69.26–75.99), 71.8 (68.33–75.27), and 72.73 (69.63–75.84) mm Hg, respectively. No statistical differences were found for SBP and DBP over time. Finally, in the overall PC group, the mean SBP values for the first, second, and third trimesters (95% CI) were 114.78 (111.39–118.16), 114.83 (111.91–117.76), and 115.78 (112.60–118.96) mm Hg, respectively. The DBP values were 67.78 (65.23–70.32), 68.89 (66.40–71.38), and 69.17 (66.60–71.73) mm Hg, respectively. There were no statistical differences in either SBP or DBP when the three groups (INF, RA, and PC) were compared in the same trimester, except for the DPB of the PC and RA groups in the first trimester, which was higher in the RA group (*p* = 0.043). In any case, the values in the RA group in that trimester were within normality.

No statistical differences were found with the maternal weight gain (kg) between the RA group (14.79 (13.19–16.40)), INF group (15.24 (14.03–16.45)), and PC group (14.86 (13.65–16.05)). No cases of gestational diabetes or preeclampsia were reported in the RA and INF groups while one case of preeclampsia and two cases of gestational diabetes mellitus were reported among women of the PC group.

The mean gestational age at birth (weeks) were 40.61 (40.08–41.14), 40.54 (39.923–41.15), and 39.43 (38.69–40.16) for the INF, RA, and PC groups, respectively. The gestational age was lower in the PC group than in the INF and RA groups (*p* = 0.021 and 0.013, respectively). In contrast to the PC group, the gestational age at birth attained by RA and INF women was always >38 weeks.

## 4. Discussion

In this study, there were differences in the vaginal *Lactobacillus* population between fertile women with a history of reproductive success and those with infertility or habitual spontaneous abortion. The lowest Nugent scores and vaginal pH values were closely linked to *Lactobacillus*-dominated communities while the contrary was linked lactobacilli depletion. Similar findings have been described previously [1,32]. Interestingly, the number of antibiotic treatments was significantly lower in the NPC group than among RA or INF women. Antibiotics have been reported as one of the main factors leading to a depletion of autochthonous vaginal lactobacilli [33]. Our data suggest that, on the one hand, the impact of antibiotherapy on vaginal lactobacilli may impair fertility or embryo implantation and, on the other hand, that this effect may be overcome by probiotic modulation of the vaginal microbiota.

The in vitro assessment of some properties of *L. salivarius* CECT 30632 revealed its ability to inhibit the growth of all the vaginal pathobionts strains used as indicators in this work. Such activity may be important to reduce the risk of genitourinary tract infections, which have been linked to poor reproductive outcomes [34]. *L. salivarius* CECT 30632 displayed a noticeable α-amylase activity and a high acidifying activity as a result of its ability to produce high amounts of L-lactic acid. These properties are very relevant for vaginal homeostasis since they promote the existence of a highly acidic vaginal pH (≤4.5), which is a feature of a *Lactobacillus*-dominated healthy vaginal ecosystem [35,36]. Although the strain was able to produce small amounts of hydrogen peroxide in vitro, the in vivo role of this compound as an antimicrobial factor in the vaginal ecosystem has been recently described as implausible since the in vitro conditions used to detect hydrogen peroxide are very different to those found within the cervicovaginal environment [37]. In addition, the antimicrobial activity of hydrogen peroxide produced by vaginal lactobacilli seems to be blocked by cervicovaginal fluid and semen [32]. This fact, together with the absence of bacteriocinogenic activity against the indicator microbes used in this work, suggest that the L-lactate may be the main compound responsible for the antimicrobial activity of the strain, similarly to that observed by other authors [34,35]. Biosurfactant production may also explain, at least partly, the antimicrobial potential of lactic acid bacteria. However, such property has not been evaluated in this study.

*L. salivarius* CECT 30632 was highly adhesive to vaginal epithelial cells and co-aggregated with the vaginal-related pathobionts tested in this work. These properties are also attractive for probiotics targeting the vagina since they allow a higher competitiveness and fitness in relation to other microorganisms that may inhabit or reach the vaginal cavity [13,38]. In addition, this strain showed a high rate of survival when confronted to the conditions that it would have to face in the human digestive tract and was highly adhesive to intestinal epithelial cells. These properties are also relevant when a probiotic strain is going to be administrated *per os* as this was the intention in the subsequent trial. Nowadays, the use of oral probiotics is fundamental in various gynaecological problems and has broad fields of application and perspectives [39].

In this work, a *L. salivarius* CECT 30632 intake led to noticeable pregnancy rates among RA and INF women, which involved significant changes in pH and Nugent score values, and in microbiological (*Lactobacillus* concentration, presence of *L. salivarius* cells and DNA) and immunological (TGF-β1, TGF-β2 and VEGF) parameters in the vaginal ecosystem.

The changes in the vaginal concentrations of VEGF, TGF-β1, and TGF-β2 may be closely associated with the efficacy of the strain. VEGF is a glycoprotein involved in endometrial angiogenesis and vasculogenesis [40,41], two processes that are essential for embryo implantation [42,43,44] and impairment of which may lead to implantation failure or abortion during the first three months of pregnancy [40,45,46,47]. TGF-β 1 and TGF-β 2 play well-known roles in the induction of active immune tolerance in mucosal sites [48,49]. There are high concentrations of both growth factors in human semen [50,51], but they require activation in an acidic environment in order to bind to cervico-vaginal cell receptors [52,53], a fact that is feasible because of the acid pH that characterized the healthy *Lactobacillus*-dominated vaginal environment [54]. Interestingly, the intake of the probiotic strain during the trial allowed not only increased vaginal TGF-β1 and TGF-β2 concentrations but, also, resulted in a significant acidification of the vaginal pH.

Safety assessment during the pilot clinical assay included the measurement of the blood pressure (both systolic and diastolic) of the INF, RA, and PC women at three sampling times (one in each pregnancy trimester). Although, overall, the use of probiotics (including some *L. salivarius* strains) in pregnant women has been demonstrated to be safe [12,55,56,57,58], a recent Cochrane revision [59] suggested a potential detrimental effect (a higher rate of preeclampsia) of some strains (*Bifidobacterium lactis* Bb12, *Lacticaseibacillus rhamnosus* GG) when used in high-risk overweight and obese pregnant women. However, such suggestion was made on the basis of a low number of studies and, among them, only one showed a potential relationship between probiotic intake and pre-eclampsia [60]. Interestingly, other trial involving the administration of the cited strains to pregnant women did not detect any deleterious change in the blood pressure of the participants through the whole pregnancy period [61]. The use of other strains by pregnant women for up to 12 weeks led to a reduction in the triglyceride levels and to beneficial effects on markers of inflammation, metabolic syndrome, and oxidative stress [62] or to a reduction of the prevalence of gestational diabetes mellitus [63]. In this study, the prolonged intake of *L. salivarius* CECT 30632 by those women of the RA and INF groups that became pregnant had no effect in relation to total weight gain during pregnancy or to the blood pressure when compared to the PC group. On the contrary, intake of the strain for the first 15 weeks of pregnancy led to a higher gestational age with no preterm deliveries reported among pregnant RA and INF women.

As a conclusion, specific vaginal strains may display a range of activities with potential to benefit reproductive outcomes, including the competitive exclusion of potentially harmful microbes that may compromise embryo implantation or fertility [64,65], contribution to vasculogenesis and angiogenesis, two processes that are required for embryo implantation [66], and immune-related activities involved in either implantation or tolerance towards the embryo [67,68]. A previous work from our group showed similar results with a different *L. salivarius* strain selected according to similar criteria [12]. This highlights the need of a careful strain-by-strain evaluation when probiotics are aimed to contribute to the fertility field, which is very appealing, having in account the limited efficacy of the treatments that are available for repetitive abortion and infertility of unknown origin [69,70].

## Figures and Tables

**Table 1:** Inhibitory activity and co-aggregation ^a^ of *L. salivarius* CECT 30632 with vaginal pathogens.

Strain	Inhibition Zone (mm)	*Co-Aggregation*
*G. vaginalis* MP14	4.5	++
*G. vaginalis* MP17	4.3	++
*G. vaginalis* MP20	4.4	++
*G. vaginalis* MP24	4.2	++
*G. vaginalis* MP29	4.3	++
*S. agalactiae* MP07	2.4	+
*S. agalactiae* MP12	2.3	+
*S. agalactiae* MP46	2.0	+
*C. albicans* MP09	3.4	++
*C. albicans* MP18	3.6	++
*C. albicans* MP31	3.0	++
*C. glabrata* MP33	2.8	++
*C. glabrata* MP37	2.6	++
*C. parapsilosis* MP36	2.9	++
*C. parapsilosis* MP48	2.7	++
*U. urealyticum* MP39	3.1	+
*U. urealyticum* MP57	3.3	+

^a^ Co-aggregation scale: +: sparsely distributed and small clumps; and ++: large and dense visible bacterial clumps.

**Table 2:** Sensitivity to antibiotics of *L. salivarius* CECT 30632.

**Antibiotic ^a^**	**GEN**	**KAN**	**STP**	**NEO**	**TET**	**ERY**	**CLI**	**CHL**
MIC value	2	128	32	4	2	0.25	0.5	2
Breakpoint ^b^	16	64 (R ^c^)	64	nr	8	1	4	4
**Antibiotic**	**AMP**	**PEN**	**VAN**	**VIR**	**LIN**	**TRM**	**CIP**	**RIF**
MIC value	0.5	0.25	>128	0.25		0.5	0.25	2
Breakpoint ^b^	4	nr	nr (R ^c^)	nr	nr	nr	nr	nr

^a^ Abbreviations: MIC: Minimal inhibitory concentration (mg/mL); GEN, gentamycin; KAN, kanamycin; STP, streptomycin; NEO, neomycin; TET, tetracycline; ERY, erythromycin; CLI, clindamycin; CHL, chloramphenicol; AMP, ampicillin; PEN, penicillin; VAN, vancomycin; VIR, virginiamycin; LIN, linezolid; TRM, trimethoprim; CIP, ciprofloxacin; RIF, rifampicin; nr, not required by EFSA [23]. ^b^ Breakpoint: microbiological breakpoints (mg/mL) that categorize *L. salivarius* as resistant (microbiological breakpoints are defined as the MIC values that clearly deviate from those displayed by the normal susceptible populations [23]. ^c^ R: the species *L. salivarius* is intrinsically resistant.

**Table 3:** Main characteristics of the women recruited in this study (*N* = 54).

		NPC *n* = 14	RA *n* = 20	INF *n* = 20	*p*-Value
**Age (years)**	Mean (95% CI)	34.6 (33.5–35.8) ^a^	39.5 (38.51–40.49) ^b^	37.95 (36.92–38.98) ^b^	<0.001 ^#^
	Range [min–max]	[28.0–45.0]	[35.0–43.0]	[35.0–44.1]	
**Weight (kg)**	Mean (95% CI)	62.4 (59.7–65.0)	69.25 (66.05–72.45)	67.55 (64.66–70.44)	0.054 ^#^
	Range [min–max]	[46.0–87.0]	[54.0–86.0]	[55.0–82.0]	
**Height (cm)**	Mean (95% CI)	166 (164–168)	166.99 (164.18–170.42)	167.80 (165.48–170.12)	0.624 ^#^
	Range [min–max]	[156–175]	[154–189]	[161–183]	
**Regularity of the menstrual cycle**	Yes, n (%)	10 (71)	9 (45)	10 (50)	0.225 *
	No, n (%)	4 (29)	11 (55)	10 (50)	
**Duration menstrual cycle (days)**	Mean (95% CI)	28.0 (27.4–28.7)	27.35 (26.73–27.97)	27.58 (26.87–28.28)	0.752 ^#^
	Range [min–max]	[25.0–32.5]	[24.0–30.0]	[24.0–30.0]	
**History of infections**					
Vaginal	n (%)	2 (14)	12 (60)	7 (35)	0.024 *
Urinary tract	n (%)	2 (14)	12 (60)	12 (60)	0.013 *
Otorhinolaryngology	n (%)	3 (21)	7 (35)	11 (55)	0.128 *
Lower respiratory tract	n (%)	2 (14)	7 (35)	7 (35)	0.344 *
Skin	n (%)	1 (7)	3 (15)	4 (20)	0.999 *
Gastrointestinal	n (%)	0 (0)	1 (5)	1 (5)	1.000
**Antibiotic usage**					
In infancy	n (%)	4 (29)	18 (90)	14 (70)	<0.001 *
In adulthood	n (%)	4 (29)	15 (75)	16 (80)	0.016 *
**History of other conditions**					
Allergies	n (%)	2 (14)	5 (25)	4 (20)	0.995 *
Food intolerance	n (%)	0 (0)	7 (35)	11 (55)	<0.001 *
Thyroid disease	n (%)	0 (0)	5 (25)	3 (15)	0.125 *

Abbreviations: NPC, non-pregnant control group; RA, repetitive abortions group; INF, infertility of unknown origin group. # One-way ANOVA tests. Different bold letters a row indicate statistically significant differences between groups (Scheffé *post hoc* comparison tests). * Freeman–Halton extension of the Fisher exact probability tests for a 2 × 3 contingency table.

**Table 4:** Baseline vaginal parameters of the women recruited in this study (*N* = 54).

		Control *n* = 14	RA *n* = 20	INF *n* = 20	*p*-Value
**pH**	Mean (95% CI)	4.53 (4.38–4.68) ^a^	5.74 (5.53–5.94) ^b^	6.03 (5.88–6.18) ^b^	< 0.001 ^#^
	Range (min–max)	(4.20–5.00)	(4.70–6.40)	(4.90–6.30)	
**Nugent score**	Mean (95% CI)	1.79 (1.27–2.30) ^a^	6.55 (5.99–7.11) ^b^	6.40 (5.90–6.90) ^b^	< 0.001 ^#^
	Range (min–max)	(0.00–4.00)	(4.00–8.00)	(4.00–8.00)	
**TGF-β1** (pg/mL)	Mean (95% CI)	4.83 (4.65–5.01) ^a^	2.46 (2.19–2.72) ^b^	2.13 (1.98–2.27) ^b^	< 0.001 ^#^
	Range (min–max)	(4.20–5.30)	(1.60–3.50)	(1.60–2.70)	
**TGF-β2** (pg/mL)	Mean (95% CI)	3.22 (3.10–3.34) ^a^	1.50 (1.35–1.65) ^b^	1.35 (1.24–1.46) ^b^	<0.001 ^#^
	Range (min–max)	(2.70–3.70)	(1.00–2.10)	(0.90–1.80)	
**VEGF** (pg/mL)	Mean (95% CI)	406.0 (322.0–490.0) ^a^	258.20 (203.76–312.64) ^a^	182.95 (136.19–229.71) ^b^	0.010 ^#^
	Range (min–max)	(1.4–929.0)	(99.0–479.0)	(69.0–433.0)	
**Lactobacilli**					
**positive women**	n (%)	14 (100)	12 (60)	7 (35)	<0.001 *
**Viable counts** (log_10_ CFU/mL) **	Mean (95% CI)	7.33 (7.15 -7.46) ^a^	4.19 (3.71–5.14) ^b^	3.65 (2.47–5.98) ^b^	<0.001 ^#^
	Range (min–max)	(6.80–7.70)	(2.10–5.20)	(2.00–5.30)	

Abbreviations: NPC, non-pregnant control group; RA, repetitive abortions group; INF, infertility of unknown origin group. # One-way ANOVA tests. Different bold letters a row indicate statistically significant differences between groups (Scheffé post hoc comparison tests). * Freeman–Halton extension of the Fisher exact probability tests for a 2 × 3 contingency table. ** Values obtained in lactobacilli-positive women.

**Table 5:** Pregnancy and successful pregnancy outcomes in the groups RA and INF after the trial.

	Group			
Outcome	RA	INF	Total (RA+INF)	Ratio (95% CI) (RA/INF)
Pregnancy (no. events/total events)	16/20	11/20	27/40	
Pregnancy effectiveness (95% CI)	80% (62.47–97.53%)	55% (33.20–76.80%)	67.5% (52.98–82.02%)	1.45 (0.92–2.29)
Successful pregnancy * (no. events/total events)	15/20	10/20	25/40	
Reproductive success (95% CI)	75% (56.02–93.98%)	50% (28.09–71.91%)	62.5% (47.50–77.50%)	1.50 (0.90–2.49)

* One woman in each group ended up having an abortion.

**Table 6:** Vaginal parameters corresponding to RA women who had a full-term pregnancy (*n* = 15) and to RA women who did not (*n* = 5) after the trial.

	Pregnancy		
	Yes (*n* = 15)	No (*n* = 5)	
Vaginal Parameter	(Mean (95% CI))	(Mean (95% CI))	*p*-Value *^#^*
**pH**			
Baseline	5.61 (5.37–5.84)	6.16 (6.04–6.39)	0.007
Post-intervention	4.44 (4.31–4.58)	5.64 (5.43–6.06)	<0.001
Change	−1.19 (−1.36–−0.86)	−0.52 (−0.62–−0.32)	<0.001
*p*-value ^†^	<0.001	0.025	
**Nugent score**			
Baseline	6.31 (5.62–7.00)	7.4 (7.12–7.95)	0.074
Post-intervention	2.25 (1.72–2.78)	6.2 (5.78–7.04)	0.001
Change	−4.2 (−4.68–−3.26)	−1.2 (−1.43–−0.75)	<0.001
*p*-value ^†^	<0.001	0.025	
**TGF-β1 (pg/mL)**			
Baseline	2.64(2.36–2.91)	1.78 (1.70–1.93)	0.002
Post-intervention	4.18125 (3.94–4.42)	2.18 (1.98–2.57)	0.001
Change	1.59 (1.31–2.15)	0.4 (0.26–0.67)	<0.001
*p*-value ^†^	<0.001	0.025	
**TGF-β2 (pg/mL)**			
Baseline	1.59 (1.43–1.74)	1.12 (0.98–1.39)	0.007
Post-intervention	2.91 (2.69–3.13)	1.34 (1.17–1.68)	0.001
Change	1.36 (1.17–1.73)	0.22 (0.03–0.59)	<0.001
*p*-value ^†^	<0.001	0.655	
**VEGF (pg/mL)**			
Baseline	296.66 (239.09–354.16)	106.60 (103.08–113.55)	0.001
Post-intervention	586.88 (479.76–693.99)	126.20 (113.63–151.04)	<0.001
Change	306.40 (233.76–449.93)	19.6 (10.10–38.37)	<0.001
*p*-value ^†^	<0.001	0.025	
**Lactobacilli presence (n (%))**			
Baseline	10 (66.66)	2 (40)	0.172 *
Post-intervention	15 (100)	3 (60)	0.052 *
Change	5 (33.33)	1 (20)	0.613 *
**Lactobacilli counts (log_10_ CFU/mL)**			
Baseline	4.08 (3.58–5.08)	4.75 (4.50–5.24)	0.685
Post-intervention	7.34 (7.08–7.85)	4.23 (3.42–5.84)	<0.001
Change	3.12 (2.51–4.33)	0.4 (0.33–0.54)	<0.001
*p*-value ^†^	<0.001	0.525	
***L. salivarius* qPCR (n (%))**			
Initial	nd	nd	nd
Post-intervention	15 (100)	2 (40)	0.035 *
***L. salivarius*** qPCR (log_10_ copies/mL) **			
Initial	nd	nd	nd
Post-intervention	7.81 (7.52–8.39)	2.25 (1.50–3.73)	<0.001

# One-way ANOVA tests with the exception of *. * Fisher exact probability test for a 2 × 2 contingency table. † One-way repeated measures ANOVA. ** Mean (95% CI) of *L. salivarius* qPCR (copies/mL) in positive samples. nd: not detected.

**Table 7:** Vaginal parameters corresponding to the INF women who had a full-term pregnancy (*n* = 10) and to the INF women who did not (*n* = 10) after the trial.

	Pregnancy		
	Yes (*n* = 10)	No (*n* = 10)	
Vaginal Parameter	(Mean (95% CI))	(Mean (95% CI))	*p*-Value *^#^*
**pH**			
Baseline	5.9 (5.68–6.12)	6.16 (6.00–6.32)	0.026
Post-intervention	4.49 (4.37–4.61)	5.88 (5.69–6.07)	<0.001
Change	−1.41 (−1.59–1.23)	−0.28 (−0.43–0.13)	<0.001
*p*-value ^†^	0.001	0.002	
**Nugent score**			
Baseline	6.2 (5.44–6.96)	6.6 (5.93–7.27)	0.571
Post-intervention	2 (1.42–2.58)	5.7 (5.04–6.36)	<0.001
Change	−4.2 (−4.90–3.50)	−0.9 (−1.44–0.36)	<0.001
*p*-value ^†^	0.001	0.027	
**TGF-β1 (pg/mL)**			
Baseline	2.26 (2.05–2.47)	1.99 (1.82–2.16)	0.070
Post-intervention	4.58 (4.39–4.77)	2.23 (2.06–2.40)	<0.001
Change	2.32 (2.16–2.48)	0.24 (0.09–0.39)	<0.001
*p*-value ^†^	0.001	0.027	
**TGF-β2 (pg/mL)**			
Baseline	1.51 (1.386–1.64)	1.19 (1.09–1.29)	<0.001
Post-intervention	2.86 (2.72–3.00)	1.33 (1.20–1.46)	<0.001
Change	1.35 (1.26–1.44)	0.14 (0.06–0.22)	<0.001
*p*-value ^†^	0.001	0.027	
**VEGF (pg/mL)**			
Baseline	261.8 (203.52–320.08)	104.1 (81.07–127.13)	<0.001
Post-intervention	734.1 (600.11–868.09)	119.7 (89.24–150.16)	<0.001
Change	472.3 (379.31–565.29)	15.6 (3.81–27.39)	<0.001
*p*-value ^†^	0.001	0.057	
**Lactobacilli presence (n (%))**			
Baseline	4 (40)	3 (30)	1.000 *
Post-intervention	10 (100)	3 (30)	0.003 *
Change	6 (60)	0 (0)	0.020 *
**Lactobacilli counts (log_10_ CFU/mL)**			
Initial	3.26 (1.77–4.60)	3.57 (1.81–5.12)	0.290
Post-intervention	6.70 (6.19–7.15)	5.4 (3.73–6.88)	0.032
Change	3.23 (2.46–3.90)	2.05 (0.05–3.82)	<0.001
*p*-value ^†^	<0.001	0.451	
***L. salivarius* qPCR (n (%))**			
Initial	nd	nd	-
Post-intervention	10 (100)	1 (10)	0.002 *
***L. salivarius*** qPCR (log_10_ copies/mL) **			
Initial	-	-	-
Post-intervention	7.05 (6.39–7.63)	4.20	-

# One-way ANOVA tests with the exception of *. * Fisher exact probability test for a 2 × 2 contingency table. † One-way repeated measures ANOVA. ** Mean (95% CI) of *L. salivarius* qPCR (copies/mL) in positive samples.

## Data Availability

The data presented in this study are available on request from the corresponding author. The data are not publicly available due to privacy restrictions.

## Supplementary Materials

The following are available online at https://www.mdpi.com/article/10.3390/nu15020362/s1: Supplementary Figure S1. History of recurrent infections and use of antibiotics in infancy and adulthood among the women recruited in this study.
