# Supplementary material for: Immunomodulation of the Vaginal Ecosystem by Ligilactobacillus salivarius CECT 30632 Improves Pregnancy Rates among Women with Infertility of Unknown Origin or Habitual Abortions
Source: Nutrients. 2023 Jan 11;15(2):362. doi: 10.3390/nu15020362 (PMC9860997; doi:10.3390/nu15020362)
Supplement: Supplementary file 1 [file nutrients-15-00362-s001.zip › nutrients-2136648-supplementary/nutrients-2136648-supplementary.pptx]

## Slide 1
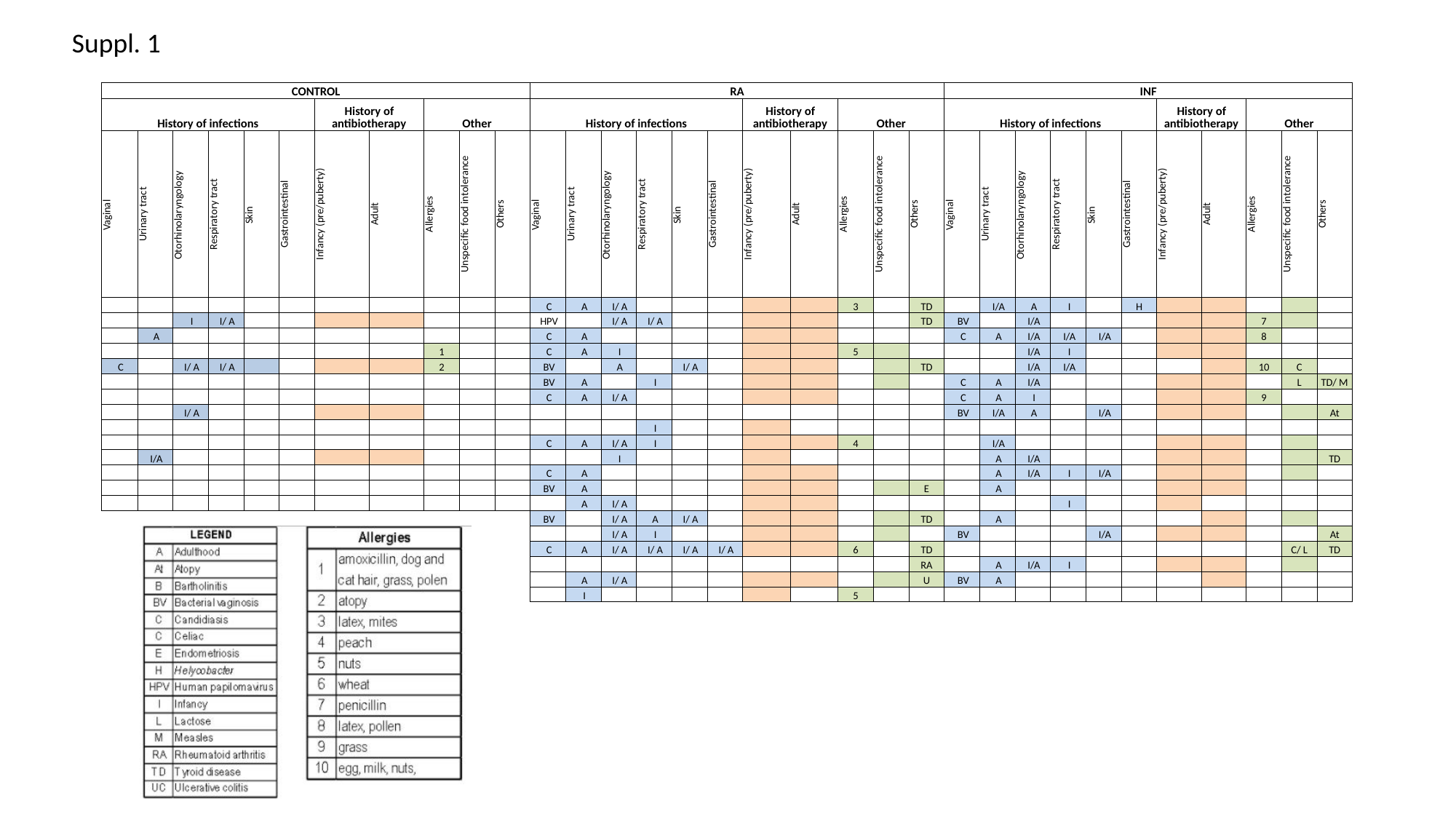

Suppl. 1
| CONTROL | | | | | | | | | | | RA | | | | | | | | | | | INF | | | | | | | | | | |
| --- | --- | --- | --- | --- | --- | --- | --- | --- | --- | --- | --- | --- | --- | --- | --- | --- | --- | --- | --- | --- | --- | --- | --- | --- | --- | --- | --- | --- | --- | --- | --- | --- |
| History of infections | | | | | | History of antibiotherapy | | Other | | | History of infections | | | | | | History of antibiotherapy | | Other | | | History of infections | | | | | | History of antibiotherapy | | Other | | |
| Vaginal | Urinary tract | Otorhinolaryngology | Respiratory tract | Skin | Gastrointestinal | Infancy (pre/puberty) | Adult | Allergies | Unspecific food intolerance | Others | Vaginal | Urinary tract | Otorhinolaryngology | Respiratory tract | Skin | Gastrointestinal | Infancy (pre/puberty) | Adult | Allergies | Unspecific food intolerance | Others | Vaginal | Urinary tract | Otorhinolaryngology | Respiratory tract | Skin | Gastrointestinal | Infancy (pre/puberty) | Adult | Allergies | Unspecific food intolerance | Others |
| | | | | | | | | | | | C | A | I/ A | | | | | | 3 | | TD | | I/A | A | I | | H | | | | | |
| | | I | I/ A | | | | | | | | HPV | | I/ A | I/ A | | | | | | | TD | BV | | I/A | | | | | | 7 | | |
| | A | | | | | | | | | | C | A | | | | | | | | | | C | A | I/A | I/A | I/A | | | | 8 | | |
| | | | | | | | | 1 | | | C | A | I | | | | | | 5 | | | | | I/A | I | | | | | | | |
| C | | I/ A | I/ A | | | | | 2 | | | BV | | A | | I/ A | | | | | | TD | | | I/A | I/A | | | | | 10 | C | |
| | | | | | | | | | | | BV | A | | I | | | | | | | | C | A | I/A | | | | | | | L | TD/ M |
| | | | | | | | | | | | C | A | I/ A | | | | | | | | | C | A | I | | | | | | 9 | | |
| | | I/ A | | | | | | | | | | | | | | | | | | | | BV | I/A | A | | I/A | | | | | | At |
| | | | | | | | | | | | | | | I | | | | | | | | | | | | | | | | | | |
| | | | | | | | | | | | C | A | I/ A | I | | | | | 4 | | | | I/A | | | | | | | | | |
| | I/A | | | | | | | | | | | | I | | | | | | | | | | A | I/A | | | | | | | | TD |
| | | | | | | | | | | | C | A | | | | | | | | | | | A | I/A | I | I/A | | | | | | |
| | | | | | | | | | | | BV | A | | | | | | | | | E | | A | | | | | | | | | |
| | | | | | | | | | | | | A | I/ A | | | | | | | | | | | | I | | | | | | | |
| | | | | | | | | | | | BV | | I/ A | A | I/ A | | | | | | TD | | A | | | | | | | | | |
| | | | | | | | | | | | | | I/ A | I | | | | | | | | BV | | | | I/A | | | | | | At |
| | | | | | | | | | | | C | A | I/ A | I/ A | I/ A | I/ A | | | 6 | | TD | | | | | | | | | | C/ L | TD |
| | | | | | | | | | | | | | | | | | | | | | RA | | A | I/A | I | | | | | | | |
| | | | | | | | | | | | | A | I/ A | | | | | | | | U | BV | A | | | | | | | | | |
| | | | | | | | | | | | | I | | | | | | | 5 | | | | | | | | | | | | | |
